# Developmental traits and life strategy of redlichiid trilobites

**DOI:** 10.1111/brv.12895

**Published:** 2022-09-12

**Authors:** Tao Dai, Xingliang Zhang, Shanchi Peng

**Affiliations:** ^1^ State Key Laboratory for Continental Dynamics and Geology Department Shaanxi Key laboratory of Early Life and Environments and Northwest University Xian 710069 China; ^2^ State Key Laboratory of Palaeobiology and Stratigraphy (Nanjing Institute of Geology and Palaeontology, CAS) Nanjing 210008 China

**Keywords:** trilobite, Cambrian, redlichiids, body plan, ontogeny, development, life cycle

## Abstract

The developmental mode of four redlichiid trilobites is summarized, based upon exceptionally well‐preserved, articulated specimens from Cambrian Series 2 (stages 3 and 4) strata in southwestern China and South Australia. These relatively complete developmental sequences indicate a balanced rate in segment increase and addition to the thorax during the meraspid phase, which might explain why most redlichiids possess micropygous body patterning during ontogeny. In addition, an analysis of the size distribution, developmental strategy, and distribution of specimen numbers at different growth stages reveals a distinct developmental strategy during the redlichiid life cycle. A relatively short pre‐holaspid and a prolonged holaspid phase in these redlichiid taxa offers insight into the developmental control and life strategy in these primitive arthropods.

## I. INTRODUCTION

Trilobites, one of the most widely recognized and well‐studied fossil groups, left a diverse and abundant fossil record throughout the Palaeozoic due to their generally biomineralized exoskeletons. During the approximately 275 million years that elapsed from the early Cambrian to the end of the Permian, they adapted to environmental changes and constantly diversified their body structures in shape, size, and trunk segment numbers. More than 20,000 species have been documented during research over two centuries, with their exoskeletal morphology, appendage structure, systematics and phylogeny well studied (e.g. Fortey & Owens, 1975, 1979; Jell, 1975; Fortey & Chatterton, 1988; Chatterton *et al*., 1990; Fortey, 1990, 2001; Chatterton & Speyer, 1997; Whittington, 1994, 1997, 2007, 2009; Adrain, 2008, 2013; Park & Kihm, 2017; Paterson, Edgecombe & Lee, 2019; Paterson, 2020). However, their ontogeny, development, and segmentation (both in the head and trunk region) within higher taxonomic groupings have long been a matter of debate and still remain unresolved.

Complete or nearly complete ontogenetic sequences based on analyses of fully articulated, well‐preserved specimens from different trilobite lineages and geological ages have been increasingly well studied. Most are considered as classic developmental models representing their respective clades, such as: the eodiscoids *Calodiscus* (Cederström *et al*., 2009), *Sinodiscus* (Dai & Zhang, 2013*a*), *Tsunyidiscus* (Dai & Zhang, 2011; Zhang & Clarkson, 2012; Dai, Zhang & Peng, 2016) and *Pagetia* (Cui *et al*., 2019); the redlichiids *Eoredlichia* (Dai & Zhang, 2013*b*), *Zhangshania* (Hou *et al*., 2017) and *Estaingia* (Holmes, Paterson & García‐Bellido, 2021*b*); the orytocephalids *Balangia* (McNamara, Yu & Zhou, 2006), *Changaspis* (Du *et al*., 2019), *Duodingia* (Hou *et al*., 2015), *Duyunaspis* (McNamara *et al*., 2006; Lei, 2016; Dai *et al*., 2017), *Oryctocarella* (Du *et al*., 2020; Dai *et al*., 2021*a*,*b*) and *Oryctocephalus* (Esteve, Zhao & Peng, 2017); the olenids *Parabolina* (Clarkson, Taylor & Ahlberg, 1997), *Ctenopyge* (Clarkson, Ahlgren & Taylor, 2003) and *Peltura* (Bird & Clarkson, 2003); and the aulacopleurids *Elrathia* (Hopkins, 2021) and *Aulacopleura* (Hughes & Chapman, 1995; Hughes, Chapman & Adrain, 1999; Hughes *et al*., 2017). These developmental sequences, with reliable ontogenetic information throughout the whole life cycle, have enabled insights into how trunk segmentation with respect to the exoskeleton was controlled and regulated, and are critical for improving our understanding of the systematic and phylogenetic relationships within this fossil arthropod group. A deeper understanding of complete growth series from different trilobite groups will provide further information for developmental and phylogenetic analyses. All the taxa referred to above displayed great diversity in morphotypes, body patterning and developmental modes, as well as a specialized trunk segmentation mode during the epimorphic phase. However, their ontogenetic sequences only constitute fewer than 30 species from the whole class.

**Fig. 1:**
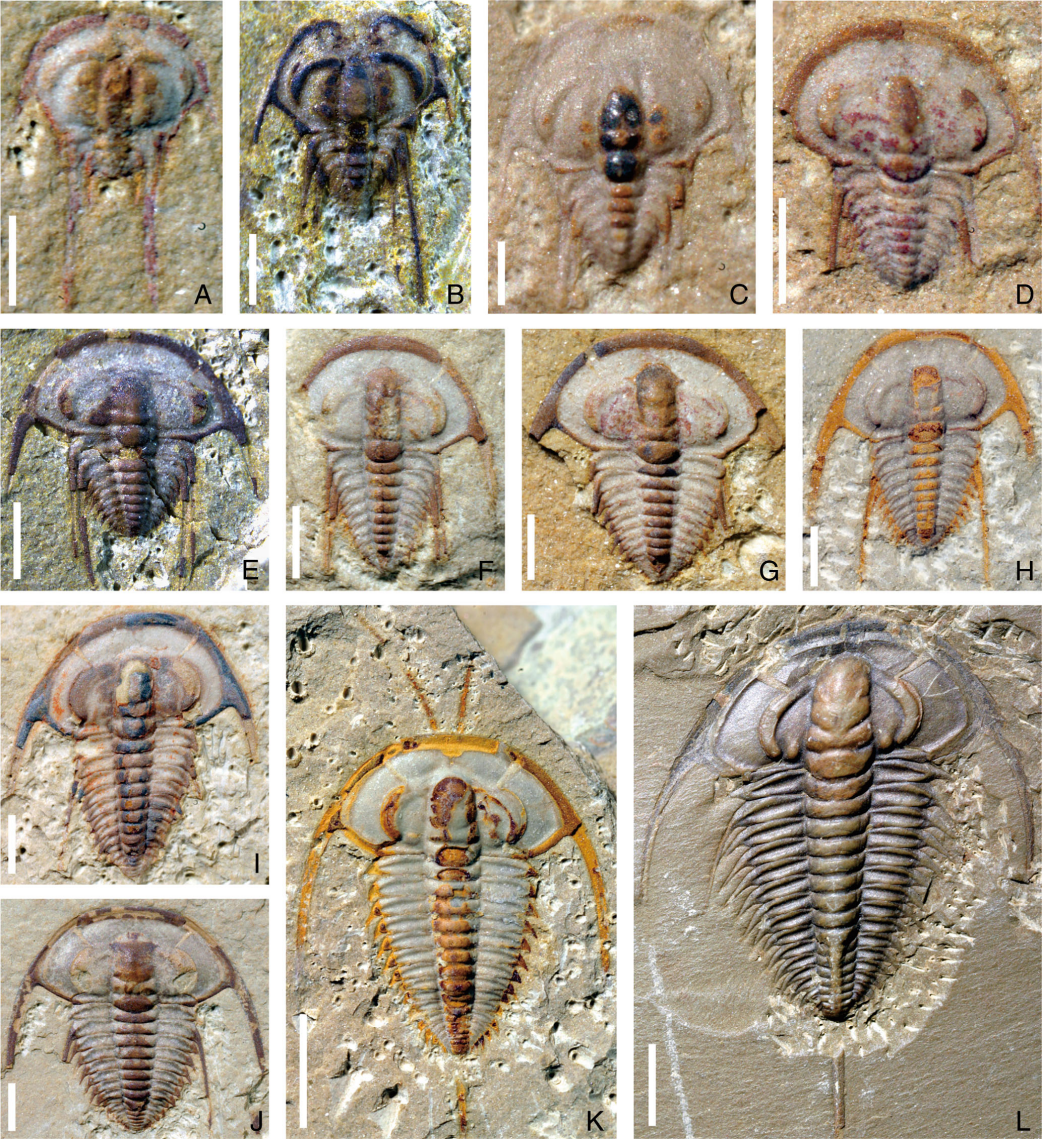
Ontogenetic sequence of *Eoredlichia intermediata* from the Cambrian stage 3, Yu'anshan Member of Heilinpu Formation, in Kunming, Yunnan Province, southwest China (from Dai & Zhang, 2013*b*). (A–J) Meraspides: (A) degree 2, NWU‐ZJX 41388; (B) degree 4, NWU‐ZJX 41326; (C) degree 5, NWU‐ZJX 41327; (D) degree 7, NWU‐ZJX 41310; (E) degree 8, NWU‐ZJX 41307; (F) degree 9, NWU‐ZJX 41316; (G) degree 10, NWU‐ZJX 41309; (H) degree 11, NWU‐ZJX 41368; (I) degree 12, NWU‐ZJX 41337; (J) degree 13, NWU‐ZJX 41356. (K, L) Holaspides; (K) stage 1, NWU‐ZJX 41355; (L) stage 2, NWU‐ZJX 41390 (NWU, Northwest University; ZJX, acronyms of specific name in Chinese). Scale bars: A–C, 0.5 mm; D–J, 1 mm; K, L, 5 mm.

**Fig. 2:**
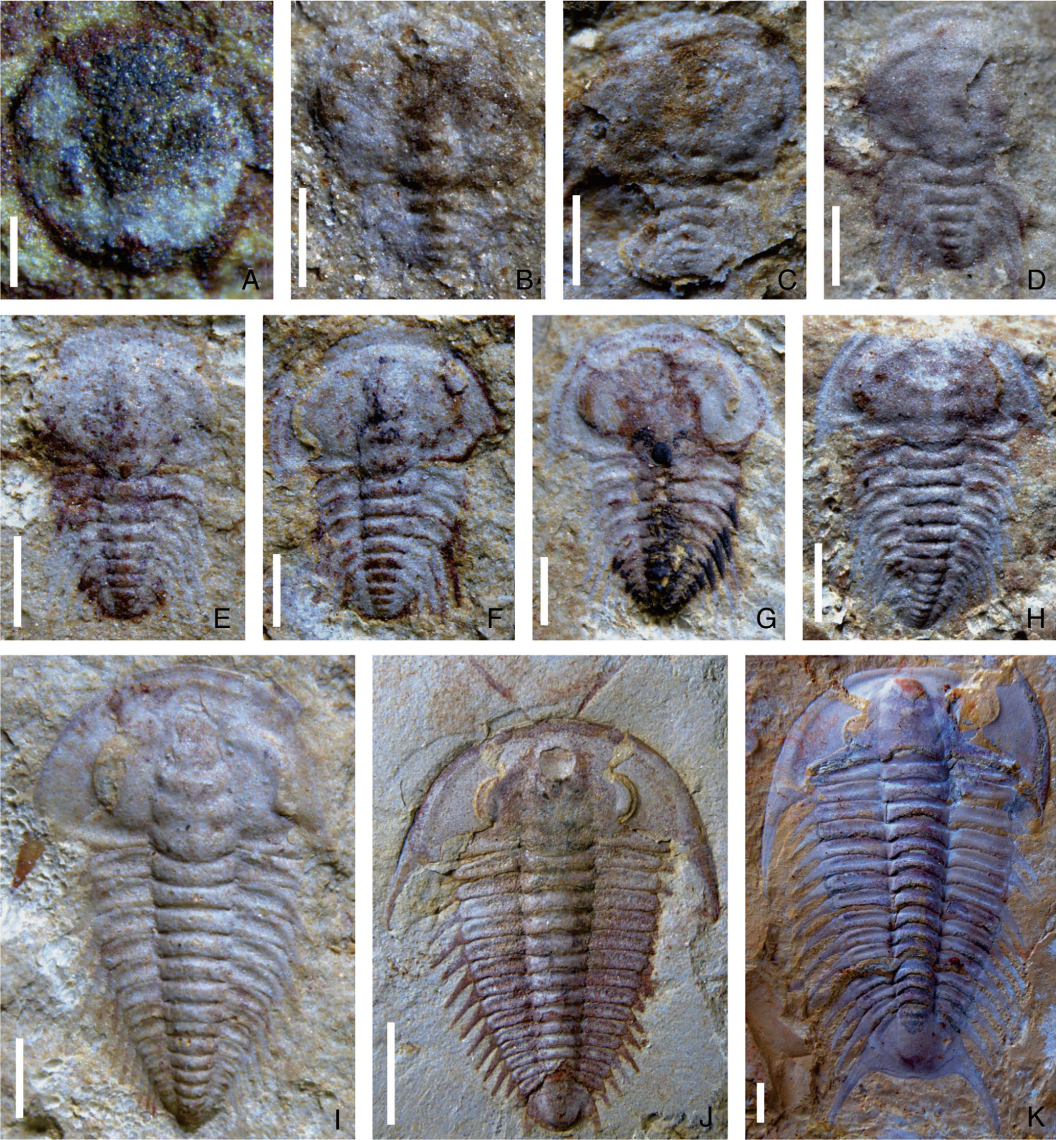
Ontogenetic sequence of *Zhangshania typica* from the Cambrian stage 3, Hongjingshao Formation in Kunming, Yunnan Province, southern China (from Hou *et al*., 2017). (A) Protaspis, YKLP 12249. (B–H) Meraspides; (B) degree 3, YKLP 12251; (C) degree 4, YKLP 12252; (D) degree 5, YKLP 12253; (E) degree 7, YKLP 12255; (F) degree 8, YKLP 12256; (G) degree 9, YKLP 12257; (H) degree 12, YKLP 12260. (I–K) Holaspides; (I) stage 1, YKLP 12262; (J) stage 3, YKLP 12264; (K) stage 4, YKLP 12266 (YKLP, Key Laboratory for Palaeobiology, Yunnan University). Scale bars: A, 0.2 mm; B–G, 0.5 mm; H, I, 1 mm; J, K, 5 mm.

**Fig. 3:**
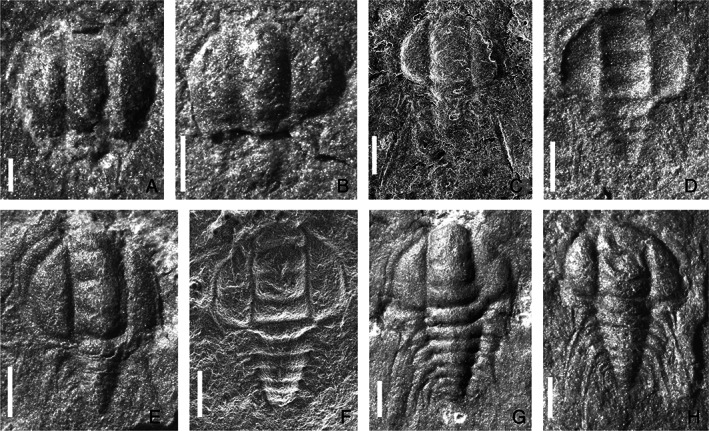
Ontogenetic sequence of *Bathynotus kueichouensis* from the Cambrian Stage 4, Kaili Formation in Danzhai County, eastern Guizhou Province, South China (from Zhang *et al*., 2022). (A) Protaspis, NWU‐GZKB 0306. (B–H) Meraspides; (B) degree 0, NWU‐GZKB 0311; (C) degree 1 or degree 2, NWU‐GZKB 0061; (D) degree 3, NWU‐GZKB 0315; (E) degree 4, NWU‐GZKB 0214; (F) degree 5, NWU‐GZKB 0330; (G) degree 6, NWU‐GZKB 0219; (H) degree 7, NWU‐GZKB 0021 (NWU, Northwest University; GZKB, acronyms of generic and specific name in Chinese). Scale bars: A, 0.2 mm; B–H, 0.5 mm.

**Fig. 4:**
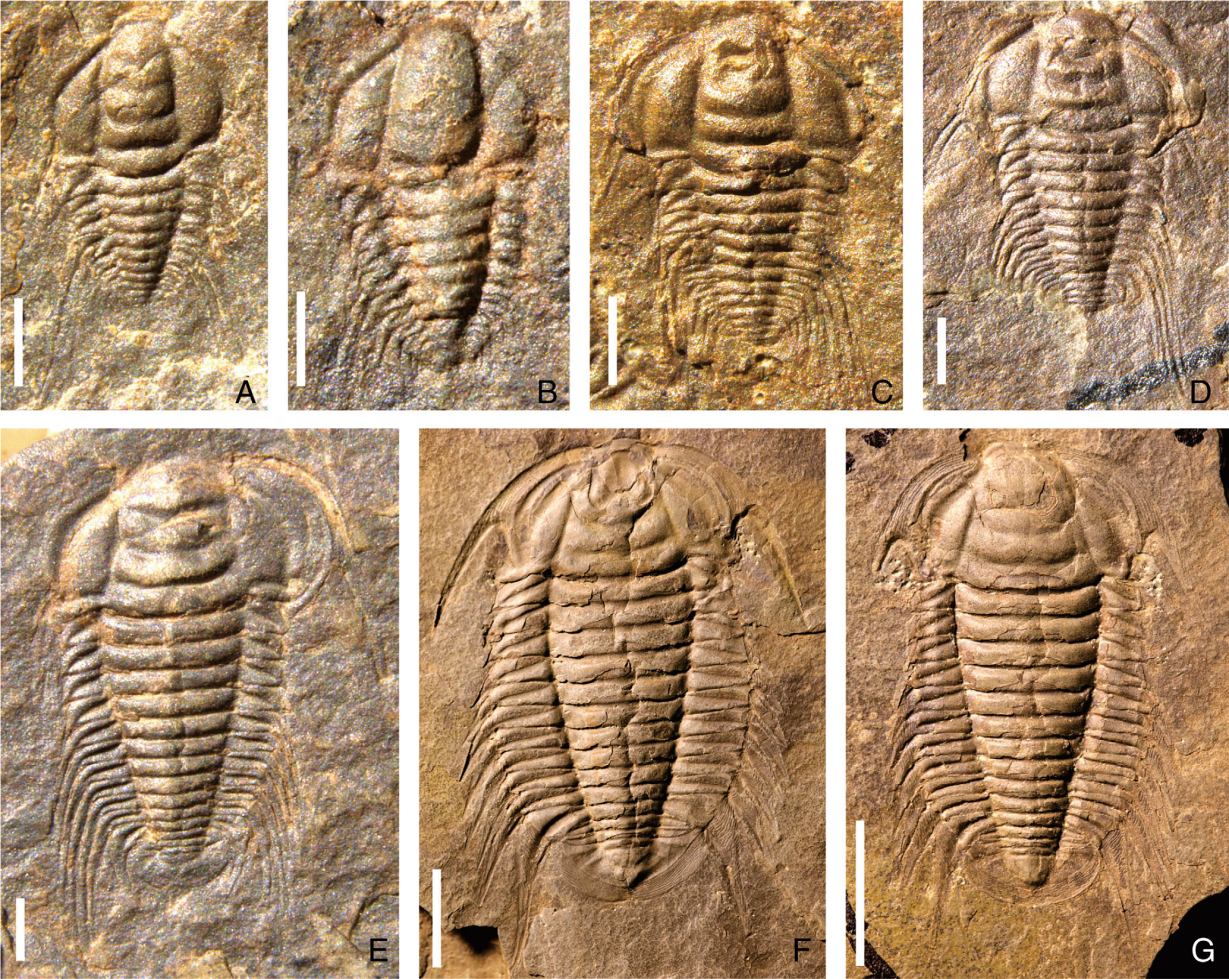
Ontogenetic sequence of *Bathynotus kueichouensis* from the Cambrian Stage 4, Kaili Formation in Danzhai County, eastern Guizhou Province, South China (from Zhang *et al*., 2022). (A–D) Meraspides; (A) degree 8, NWU‐GZKB 0061; (B) degree 9, NWU‐GZKB 0092; (C) degree 10, NWU‐GZKB 0037; (D) degree 11, NWU‐GZKB 0158. (E–G) Holaspides; (E) stage 1, NWU‐GZKB 0085; (F) stage 2, NWU‐GZKB 0326, (G) stage 2, NWU‐GZKB 0320 (NWU, Northwest University; GZKB, acronyms of generic and specific name in Chinese). Scale bars: A–E, 1 mm; F–G, 1 cm.

**Fig. 5:**
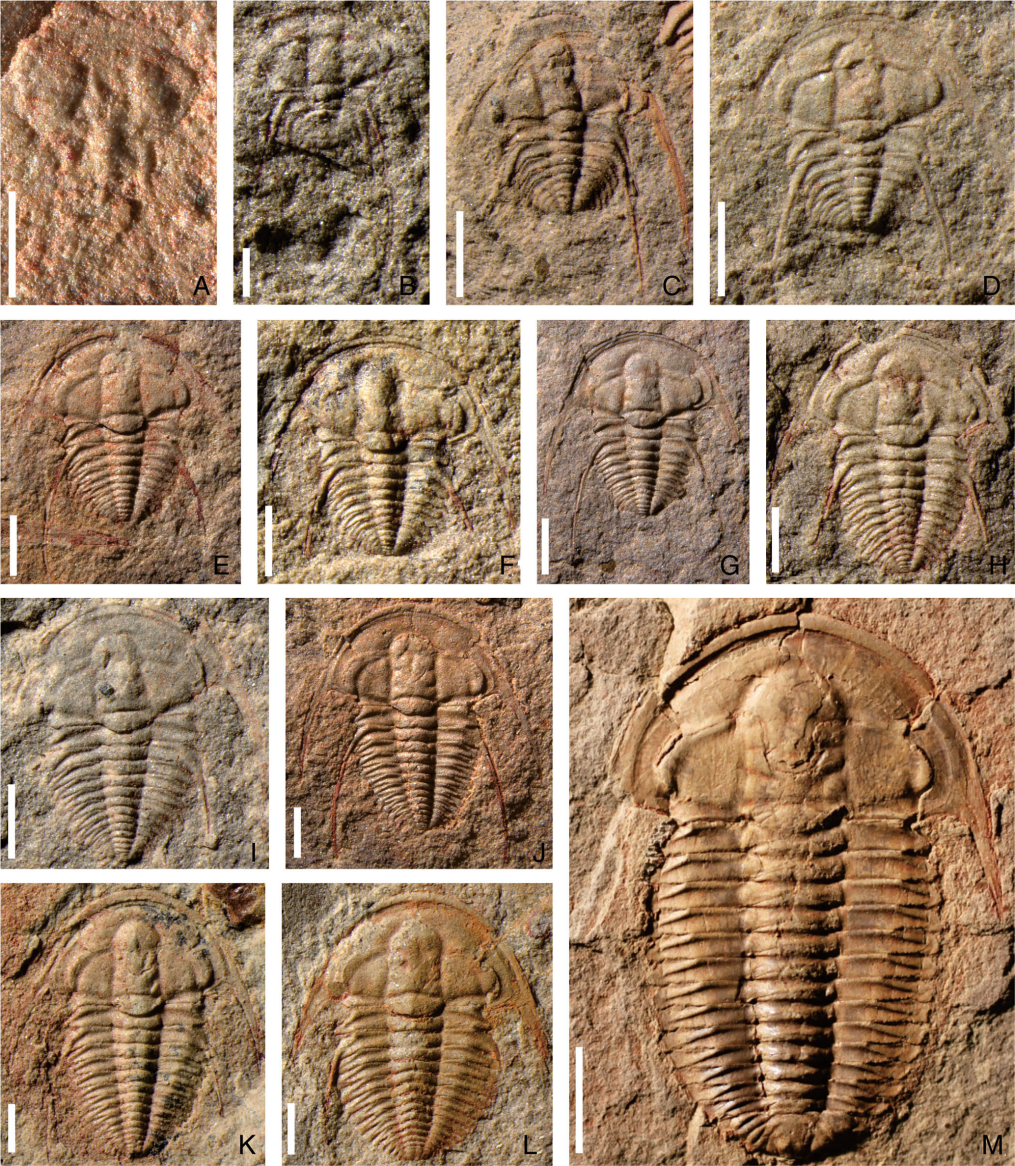
Ontogenetic sequence of *Estaingia bilobata* from the Cambrian stage 4, Emu Bay Shale, Kangaroo Island, South Australia (from Holmes *et al*., 2021*b*). (A) Protaspis, SAM P57604. (B–K) meraspides; (B) degree 1, SAM P15274; (C) degree 4, SAM P52791; (D) degree 5, SAM P57613; (E) degree 6, SAM P44572; (F) degree 7, SAM P46219; (G) degree 8, SAM P15472; (H) degree 9, SAM P57626; (I) degree 10, SAM P57640; (J) degree 11, SAM P44637; (K) degree 12, SAM P45956. (L, M) Holaspides; (L) early stage, SAM P57661; (M) late stage, SAM P15465. (SAM P, South Australian Museum, Adelaide, palaeontological collection). Scale bars: A, B, 0.5 mm; C–L, 1 mm; M, 5 mm.

The excellent fossil record of redlichiid exoskeletons, coupled with information on their non‐biomineralized tissues, permits analysis of how redlichiid taxa organized, developed and modified their body structure during various evolutionary processes (e.g. Whittington, 1990; Hou *et al*., 2009; Paterson *et al*., 2007; Webster, 2007, 2009; Esteve, Hughes & Zamora, 2013; Esteve, 2014; Geyer, 2015; Geyer & Vincent, 2015; Laibl, Esteve & Fatka, 2016; Holmes, Paterson & García‐Bellido, 2020; Yang *et al*., 2021). Ontogenetic information from a large variety of members of the order Redlichiida has been published (e.g. Kobayashi & Kato, 1951; Dai & Zhang, 2012*a*; Paterson & Edgecombe, 2006; Webster, 2007, 2009; Holmes *et al*., 2021*a*; Laibl, Maletz & Olschewski, 2021), but cases with multiple articulated specimens representing the full (or nearly full) range of developmental stages are still very rare. As far as we are aware, complete ontogenetic sequences based upon articulated specimens are available for only the redlichiids *Eoredlichia intermediata* (Fig. 1) (see Dai & Zhang, 2013*b*), *Zhangshania typica* (Fig. 2) (see Hou *et al*., 2017), *Bathynotus kueichouensis* (Figs 3 and 4) (see Zhang *et al*., 2022) and the ellipsocephaloid *Estaingia bilobata* (Fig. 5) (see Holmes *et al*., 2021*b*), all of which are assigned to the suborder Redlichiina (Adrain, 2011).

Herein, we summarize and compare the developmental sequence of these redlichiid trilobites from Cambrian Series 2 (Stage 3 and 4) strata in southwestern China and South Australia. Their trunk segmentation schedules largely exhibit a balanced relationship in segment increase and addition to the thorax during meraspid ontogeny. We term the pattern of redlichiid development described herein as ‘Lu's ontogenesis’ to commemorate the contributions of Yanhao Lu (1913–2000) to the study of trilobite systematics and stratigraphy, as well as his first ontogenetic description of redlichiid trilobites from Chinese material (Lu, 1940). A developmental comparison of growth dynamics regarding segment generation and degeneration among these taxa from different ages and families could provide us with an understanding of how developmental processes evolved within the Redlichiida. In addition, considerable evidence from the fossil record regarding their life cycles is also discussed. Based on the size distribution, developmental strategy and number of specimens at different growth stages, changes in the duration of the pre‐holaspid and holaspid phases may have particular relevance to understanding Cambrian arthropod evo‐devo.

## II. DEVELOPMENTAL TRAITS OF REDLICHIIDS

### (1) The stability of trunk segment development

**Fig. 6:**
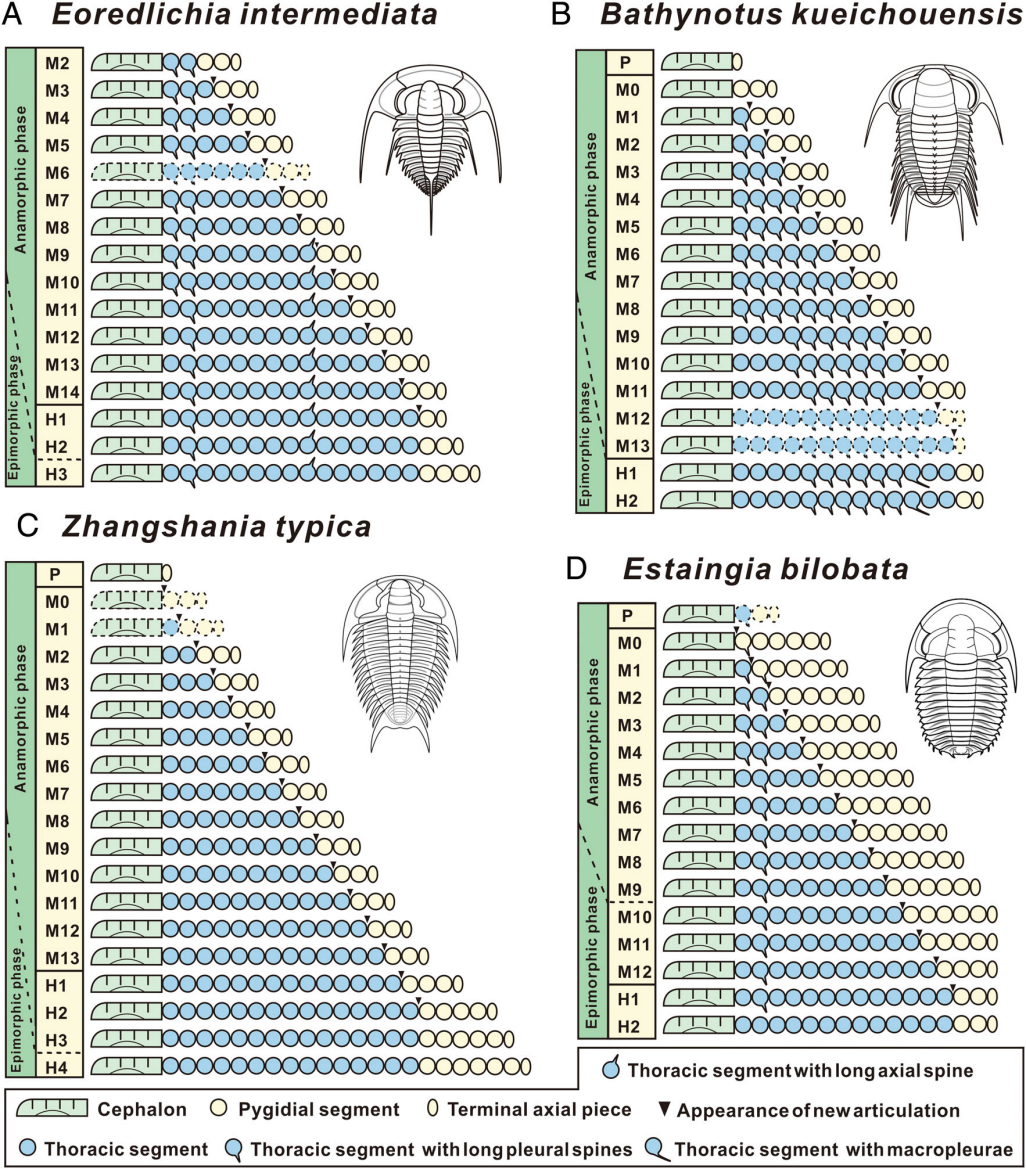
Trunk segmentation schedule of redlichiid trilobites: (A) *Eoredlichia intermediata*; (B) *Bathynotus kueichouensis*; (C) *Zhangshania typica*; (D) *Estaingia bilobata*. P, protaspid period; M, meraspid period; H, holaspid period; dotted lines represent hypothesized forms for which no material is available; green, cephalic region; blue, thoracic region; yellow, pygidial region.

For these micropygous trilobites with multiple trunk segments, the entire ontogenetic sequence from protaspid to holaspid period has been reconstructed on the basis of articulated specimens in *Z. typica* (Hou *et al*., 2017), *B. kueichouensis* (Zhang *et al*., 2022), and *E. bilobata* (Holmes *et al*., 2021*b*), and from meraspid to holaspid period in *E. intermediata* (Dai & Zhang, 2013*b*). These ontogenetic series enable subtle sequential variations in the morphological characteristics between adjacent instars to be visualized (Figs 1, 2, 3, 4, 5). The reconstructed trunk segmentation schedules (Fig. 6) allow a comparative study of the developmental patterns involving trunk segment generation and articulation, as well as a comprehensive analysis of the evolution of such developmental traits in the Redlichiida.

#### (a) Protaspis

Morphological variation within the protaspid phase in different groups of the order Redlichiida (specifically in the suborder Redlichiina) has been described for approximately 10 species (e.g. Whittington, 1957; Šnajdr, 1958; Pocock, 1970; Öpik, 1975; Palmer & Rowell, 1995; Dai & Zhang, 2012*a*,*b*; Hou *et al*., 2017). Only a few well‐preserved redlichiid protaspides have been recorded and described with multiple growth stages recognized. This careful work resulted in identification of protaspides in several families and the ontogenetic trajectories during the earliest post‐embryonic period. The best examples are for *Hydrocephalus carens* and *Eccaparadoxides pusillus* from Miaolingian Series (Drumian) in the Skryje‐Týřovice Basin of the Barrandian area, Czech Republic (Laibl, Esteve & Fatka, 2017; Laibl, Cederström & Ahlberg, 2018), *Metaredlichia cylindrica* and *Estaingia sinensis* from the Cambrian Series 2 (Stage 3) Shuijingtuo Formation in Hubei Province, South China (Dai & Zhang, 2012*a*,*b*), *Redlichia cf. versabunda* from the Cambrian Series 2 (upper Stage 4) Ramsay Limestone of Yorke Peninsula in South Australia (Holmes *et al*., 2021*a*), and phosphatized specimens currently assigned as ‘genus and species indeterminate 1’ from the Cambrian Series 2 (Stage 3) Shuigoukou Formation in Henan Province, China (Zhang & Pratt, 1999) and Shuijingtuo Formation in Shaanxi Province, China (Zhang & Clarkson, 2012), respectively.

**Fig. 7:**
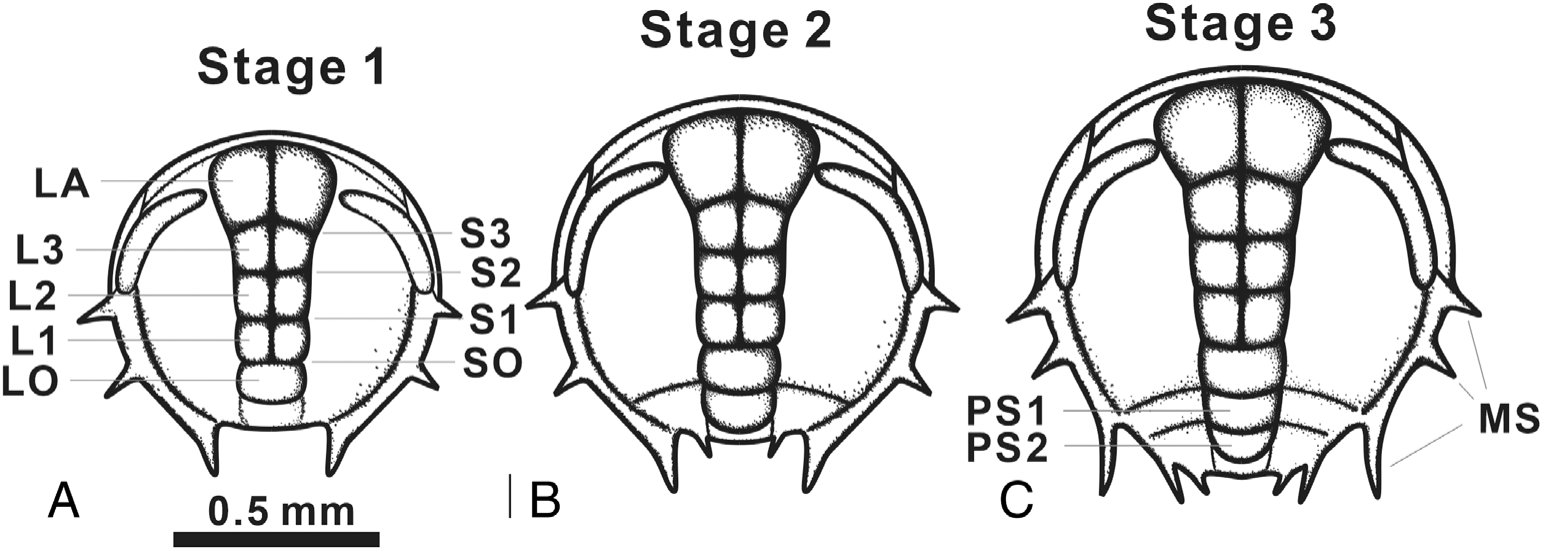
Three protaspid stages of *Metaredlichia* (see Dai & Zhang, 2012*a*), illustrating a generalized protaspid morphology of redlichiid trilobites. (A) stage 1; (B) stage 2, with appearance of the protopygidium; (C) stage 3, with appearance of one more protopygidial segment. L, protoglabellar lobes; LO, occipital ring; L1–L3, the first to the third protoglabellar lobe; LA, anterior glabellar lobe. S, protoglabellar furrow; SO, occipital furrow; S1–S3, the first to the third protoglabellar furrows. PS, protopygidial segment. MS, marginal spine.

These protaspides share many morphological characteristics, such as a generally sub‐circular outline, an anteriorly expanded axis composed of at least five segments (LO–LA) with four glabellar lobes (L1–LA) often bilobed, an anterior lobe (LA) distinctly wider and expanded forward, and three pairs of marginal spines (Fig. 7). No sub‐stages initially were recognized within the protaspides of *E. intermediata* (see Lu, 1940), *Z. typica* (Fig. 2A), *B. kueichouensis* (Fig. 3A) and *E. bilobata* (Fig. 5A) due to an extremely small quantity of specimens available. However, according to Dai & Zhang (2012*a*,*b*), Laibl *et al*. (2017, 2018) and Holmes *et al*. (2021*a*), the redlichiid protaspides can be subdivided into several growth stages based on the appearance of a shallow furrow and the number of protopygidial segments (Fig. 7), indicating that somitogenesis also occurred during the protaspid phase of various redlichiid taxa.

Thus, the protaspid phase of redlichiids typically embraced a small number of instars with different size ranges and trunk segments. Various morphological structures or ontogenetic events, such as the appearance of facial sutures, distinct axial furrows, connective and hypostomal sutures, a shallow furrow differentiating the protocranidium from the protopygidium, and the timing of a major metamorphosis from non‐adult‐like to adult‐like, have been used as dividing markers. However, these characters are not developmentally homologous within all trilobite taxa due to inconformity in their occurrence time (Chatterton & Speyer, 1997; Park & Choi, 2011), and may vary with different morphotypes among groups or even within closely related groups. Consequently, because of such difficulties with homology, former terminology, such as ‘anaprotaspis’ and ‘metaprotaspis’, which were differentiated by the appearance of a shallow furrow between the protocranidium and protopygidium (Beecher, 1895), has been abandoned (see Chatterton & Speyer, 1997). However, the addition of segments to the protopygidium has been found across taxa and consequently still seems to be a valid criterion for examining growth rates among different redlichiid protaspides.

#### (b) Meraspis

Since the number of protopygidial segments accumulated to the end of the protaspid phase is not necessarily homologous among clades before the first articulation between the head and trunk, the number of trunk segments at the onset of the meraspid phase could be variable. This lack of association between the onset of the meraspid phase and a specific number of trunk segments also indicates that the first meraspid instar may not be ontogenetically homologous even within closely related taxa. The meraspid period can be divided into a series of degrees, corresponding to sequential segment release from the anterior portion of the pygidium as freely articulating thoracic tergites (Fig. 6). Note that this does not necessarily correspond to the number of meraspid instars, due to the inconsistent segment generation and liberation during each moulting event.

Unlike most euarthropods where the caudal plate is composed of a single segment, the majority of trilobites had a caudal plate that was made up of segments rigidly conjoined to each other. Although all trunk segments were generated at a subterminal growth zone (at the anterior end of the pygidium) throughout postembryonic ontogeny, not all newly generated segments were released into the thorax. The rate of segment expression (in the subterminal growth zone) and segment release (into the thorax) determined the number of trunk segments and segment allocation between the thoracic and pygidial region, which can vary greatly among species and even higher‐level taxa. Cases where meraspid degrees do represent sequential instars with a 1:1 relationship between segment increase and release at each moult can be found in redlichiids (see below). By contrast, many other clades clearly show more than one instar per meraspid degree, such as the eodiscoids (Cederström *et al*., 2009; Dai & Zhang, 2011, 2013*a*; Zhang & Clarkson, 2012; Dai *et al*., 2016), orytocephalids (Hou *et al*., 2015; Dai *et al*., 2017, 2021*b*; Du *et al*., 2020) and aulacopleurids (Hughes & Chapman, 1995); while others presumably added more than one thoracic segment per instar, such as the emuellid *Balcoracania* which had up to 103 thoracic segments (Paterson & Edgecombe, 2006; Hughes, 2007).

In the four redlichiid species considered herein, all meraspid degrees (Figs 1A–J, 2B–H, 3B–H, 4A–D, and 5B–K) can be differentiated on the basis of the number of thoracic segments and are characterized by an approximately 1:1 balanced segment formation and addition to the thorax (except for the late meraspid period of *E. bilobata*; Fig. 6D). Thus, this developmental pattern occupied all or most of their meraspid phase. Consequently, we postulate that at the transition between each meraspid degree, as a new segment was added sub‐terminally at the pygidium, one segment was released simultaneously from the anterior portion of the meraspid pygidium into the thorax as a thoracic segment. With an extended equilibrium phase (Simpson *et al*., 2005) during the entire meraspid period, all these taxa show a small pygidium with a relatively stable segment number. This size conservation of the meraspid pygidium thus arises from a match between the expression of a new segment in the subterminal growth zone of the pygidium and the release of the previous segment into the thorax. This suggests that all (or most) redlichiid taxa with a micropygous body pattern probably increased their thoracic segment numbers through a similar developmental mode of segment increase and release.

In addition to their accumulation and release, which can be traced stage by stage, the trunk segments could also vary in morphology during meraspid ontogeny among different redlichiids. In the thoracic region, the segments usually display either a homonomous condition, with all tergites showing a similar shape and differing only in size, or a heteronomous condition with individual segments showing different morphologies or more than one segment type appearing in batches. For example, some redlichiid taxa developed one (or more) individual segments that differed from others in unique ways, such as in *E*. *intermediata*, *E. bilobata* and *Eccaparadoxides pradoanus* (Esteve, 2014) which had one or two pairs of long pleural spines on T1 (the first thoracic tergite) and/or T2, *Myopsolenites altus* (Geyer & Vincent, 2015) and *B. kueichouensis* which had one pair of macropleurae in T11, and *Z. typica* with extremely long pygidial spines that appeared from late meraspid ontogeny. Some taxa developed sequences of trunk segments into distinctive regions of similar segments that differed from those in other regions, such as the protrunk and opisthotrunk found in many olenelloids and emuellids.

#### (c) Holaspis

The equilibrium between somitogenesis and tagmosis observed during the meraspid phase seemed to be broken at the onset of the holaspid phase (Figs 1K,L, 2I–K, 4E–G, and 5L,M), and is followed by a marked variation in growth mode of the pygidial segments among the four species (Fig. 6). In *E*. *intermediata* and *B. kueichouensis*, the pygidia in H1 probably had only two segments, one fewer than those of the final meraspid degree (i.e. M14 and M11, respectively); similarly, the pygidium in H1 of *E. bilobata* had three segments, one fewer than that of the last meraspid degree (M12); while in *Z. typica*, the holaspid pygidium in H1 had one more segment than that of M13. The number of segments in the holaspid pygidium continued to increase in some species, with zero, two, three and zero segments added in *B. kueichouensis*, *E. intermediata*, *Z. typica* and *E. bilobata*, respectively. More than one holaspid growth stage can be identified according to the addition of new segments (*E*. *intermediata* and *Z. typica*), as well as the expression of other morphological traits including the size of the pleural spine of T11 in *B. kueichouensis* and morphological variation in the pygidial spines in *Z. typica*. Therefore, as individuals entered the holaspid period, segment generation probably continued in the pygidial exoskeleton, but somitogenesis was apparently not coordinated with moulting events.

Unlike the preceding meraspid equilibrium phase, the onset of the holaspid phase of *E. intermediata* is characterized by a decrease of one segment, followed by moults in which the number of holaspid pygidial segments increased (Fig. 6A); *B. kueichouensis* and *E. bilobata* are also characterized by a decrease of one segment, here followed by moults in which the number of holaspid pygidial segments remained stable (Fig. 6B, D). Such a growth pattern implies a temporary cessation of new trunk segment production at the onset of the holaspid stage for one instar, with renewed segment generation in later instars followed by final cessation of segment generation at the onset of epimorphosis (Hou *et al*., 2017). By contrast, when entering the holaspid phase, *Z. typica* (Fig. 6C) is characterized by a two‐segment increase, followed by moults in each of which the number of holaspid pygidial segments increased. The moult transitioning into the holaspid phase of *Z. typica* thus involved more than a single segment, and the pygidium apparently intermittently accumulated additional segments during the early holaspid period (Hou *et al*., 2017). In these redlichiids, accordingly, although the maximum number of thoracic segments had been reached, holaspid segmentation exhibited markedly different modes.

#### (d) Comparison with other trilobites

The consistent, equilibrium phase of segment increase and release observed in these redlichiid meraspides contrasts with the situation known in some other trilobites, in which the rate of segment increase did not equate with thoracic segment numbers during ontogeny. For example, the eodiscoids (Jell, 1975; Dai & Zhang, 2011, 2013*a*; Zhang & Clarkson, 2012; Dai *et al*., 2016, 2017), oryctocephalids (Hou *et al*., 2015; Lei, 2016), ptychopariids (Stubblefield, 1926; Fortey & Owens, 1991; Shen *et al*., 2014) and aulacopleurids (Hughes *et al*., 2017) displayed a more complex development of the trunk segmentation (especially during the anamorphic phase) than the redlichiid taxa referred to above. Multiple morphs within individual meraspid degrees have been identified in these clades, perhaps due to intraspecific variation in trunk segmentation (Dai *et al*., 2017), polymorphism in the pattern of segment release (Hughes & Chapman, 1995; Fusco, Hong & Hughes, 2014), or intrapopulation phenotypic variation in the degree of segment expression (Hou *et al*., 2015).

Unlike micropygous redlichiids, all these taxa have an isopygous or subisopygous body patterning, with a relatively large pygidium containing more segments throughout ontogeny. A more complex condition in the segment exchange between the thorax and pygidium makes the trunk segmentation of these groups seemingly ‘irregular’ (see Dai *et al*., 2021*a*,*b*), and muddied our understanding of trilobite segmentation in the context of their lifestyle and functional morphology (see Dai *et al*., 2019). It is likely that different body patterning associated with the shape and size of the head and trunk could play a key role in trilobite development and provide insights into how various trilobites controlled their body morphology (including size, shape and segment number in both the thorax and caudal plate) through different rates of segment production and liberation.

### (2) Hemianamorphic development

The ontogenetic sequence of all trilobite taxa represents a two‐phased postembryonic development, classified as hemianamorphic development (Enghoff, Dohle & Blower, 1993), as seen in a majority of trilobite cases for which a developmental series has been documented (McNamara, Yu & Zhou, 2003; McNamara *et al*., 2006; Simpson *et al*., 2005; Hughes, Minelli & Fusco, 2006; Park & Choi, 2009; Crônier, Bignonignon & Francois, 2011; Park & Kihm, 2015; Esteve *et al*., 2017; Du *et al*., 2020). This developmental mode, which includes an anamorphic phase (i.e. a final and fixed number of body segments is reached after a series of moults) and an epimorphic phase (no further segment increase during subsequent growth and moulting events), is widespread throughout the postembryonic development of many arthropod clades and many extant arthropods (Hughes & Chapman, 1995; Hughes, 2007).

A series of growth patterns can be derived through a comparison between the onset and termination of the anamorphic and epimorphic phases and the thoracic segment accretive and invariant phases (meraspid–holaspid transition). These are termed protomeric, synarthromeric, hypoprotomeric, protarthrous and euprotomeric development (Hughes *et al*., 2006). Using these five modes to describe a ‘space of segmentation schedules’ for trilobite trunk development, a database was constructed including 65 species for which growth increments between successive instars had been published. In these samples, which spanned a wide taxonomic range and temporal diversity among closely related taxa, the trunk segmentation of 35 trilobite species were assigned to the five developmental modes. Their results showed that these developmental modes varied widely across the Trilobita, even at lower taxonomic levels.

Unfortunately, no redlichiid species was listed in this database. As a complement to this database, therefore, below we classify the four redlichiids considered herein according to the same five developmental modes.

1. The trunk development of *E. intermediata* and *Z. typica* is of the protarthrous type, since the onset of the holaspid phase significantly preceded the onset of the epimorphic phase (Fig. 6A, C). From our current understanding, the protarthrous developmental pattern seems widespread not only in the earliest clades, such as Redlichiidae (Dai & Zhang, 2013*b*), Gigantopygidae (Hou *et al*., 2017), Calodiscidae (Cederström *et al*., 2009; Dai & Zhang, 2013*a*), Tsunyidiscidae (Dai & Zhang, 2011; Zhang & Clarkson, 2012; Dai *et al*., 2016), Eodiscidae (Cui *et al*., 2019), and Oryctocephalidae (Hou *et al*., 2015; Dai *et al*., 2017), but also in other later clades such as Shumardiidae (Stubblefield, 1926), Dolichometopidae (Robison, 1967), Aulacopleuridae (Hughes & Chapman, 1995; Hughes *et al*., 1999, 2017), and Dalmanitidae (Drage, Laibl & Budil, 2018). It appears that termination of thoracic segment articulation preceded termination of trunk segment expression in various groups from different ages, and thus, this developmental pattern is not exclusive to those Cambrian taxa.
2. The trunk development of *E. bilobata* conforms to the protomeric (specifically hypoprotomeric) type, with the epimorphic phase being reached before the holaspid period, and the onset of articulation preceding that of the epimorphic phase (Holmes *et al*., 2021*b*). This pattern is largely found in phacopids, proetids and lichids (Hughes *et al*., 2006).
3. In *B. kueichouensis*, H1 appears to be accompanied by the liberation of two micropleural segments, but the holaspid pygidium then continued with only two segments, and no further segment release in later growth stages (H2) (Fig. 6B). The change from a segment accretive to an invariant phase thus coincides with the meraspid–holaspid transition. Accordingly, the onset of the holaspid phase presumably coincided with the epimorphic phase, meaning that the development of *B. kueichouensis* can be described as a synarthromeric pattern similar to that found in post‐Cambrian taxa such as Ordovician pliomerids (Simpson *et al*., 2005) and Devonian phacopids (Crônier, 2010; Crônier *et al*., 2011). Thus, this developmental mode was not only found in post‐Cambrian taxa, but also in earlier clades, and accordingly is not exclusive to groups from particular ages.

The characteristics of these developmental modes need to be tested further through exploration of additional high‐quality ontogenetic series with more complete and articulated specimens. An expanded data set needs to be created to collect more information, particularly on the characteristics of diversified development of articulations in Cambrian redlichiids, and to evaluate how such diversity evolved among derived species.

## III. LIFE CYCLE OF REDLICHIIDS

The postembryonic development of trilobites is divided into three main phases, the protaspid, meraspid, and holaspid periods, all of which can be subdivided into a number of stages that are usually synonymous with moults or instars (Chatterton & Speyer, 1997). Although there are no data regarding how many moults occurred during trilobites' life cycles or on their lifespans, considerable variation must exist among clades and even among species. Presumably, trilobites of large size or with many thoracic (or trunk) segments moulted a greater number of times and had a longer lifespan than those of small size or with fewer thoracic segments. For example, according to Jell (1975) and Cui *et al*. (2019), *Pagetia* had only two thoracic segments and at least nine instars (two protaspides, four meraspides and three holaspides) and thus must have moulted at least eight times during its life cycle. By a similar reasoning, most other eodiscoids, such as *Calodiscus* (Cederström *et al*., 2009), *Sinodiscus* (Dai & Zhang, 2013*a*), *Tsunyidiscus* (Dai & Zhang, 2011; Zhang & Clarkson, 2012; Dai *et al*., 2016), had three thoracic segments and exhibited more than nine instars and thus must have moulted more than nine times during their life cycles. By contrast, some Cambrian redlichiid species had a large number of thoracic segments: *Balcoracania dailyi* adults had up to 103 thoracic segments (Paterson & Edgecombe, 2006), and may have moulted several tens of times or even a hundred times during its life cycle.

Furthermore, we do not have reliable evidence regarding the proportion of the life cycle taken up by the different developmental phases during a trilobite's life cycle. Speyer & Chatterton (1989) and Chatterton & Speyer (1997) provided hypotheses on the distribution of the developmental stages during a trilobite's life, suggesting that the protaspid, meraspid, and holaspid periods occupied approximately one‐eighth, a quarter and half of the life history, respectively. Chatterton & Speyer (1997) argued that a substantial part of trilobite growth, from less than 25% to as much as 30–40%, probably took place during the meraspid period. Given the developmental traits summarized above, we conclude that these proportions might be variable within groups. Below we consider the likely increments between growth stages within the life cycle of our four redlichiids from three perspectives: their size distribution, developmental strategy and the distribution of specimens at different growth stages.

### (1) Size distribution

**Fig. 8:**
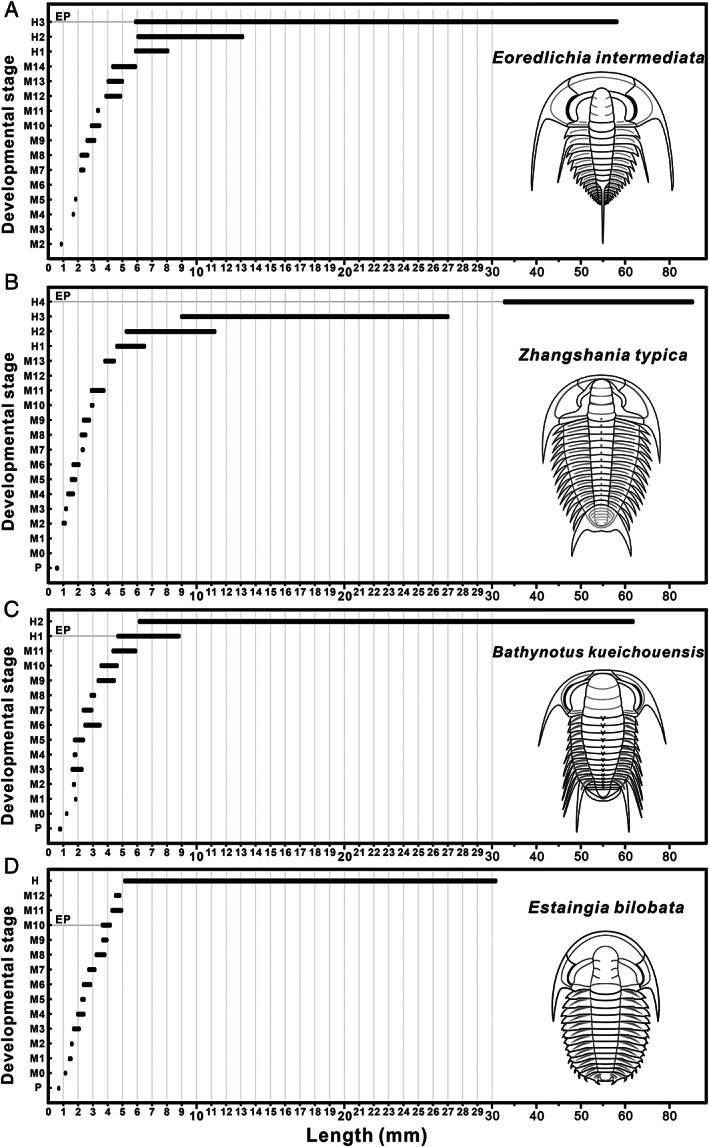
Exoskeleton length (from anterior cranidial margin to posterior pygidial margin, excluding the length of the axial spine or pygidial spine) distribution for each developmental stage in the life cycles of four redlichiid trilobites, showing a relatively shortened pre‐holaspid phase with a rapid rate of segment production and liberation and then a prolonged holaspid period with rapid enlargement in body size. (A) *Eoredlichia intermediata*. (B) *Zhangshania typica*. (C) *Bathynotus kueichouensis*. (D) *Estaingia bilobata*. EP, onset of epimorphic phase.

**Fig. 9:**
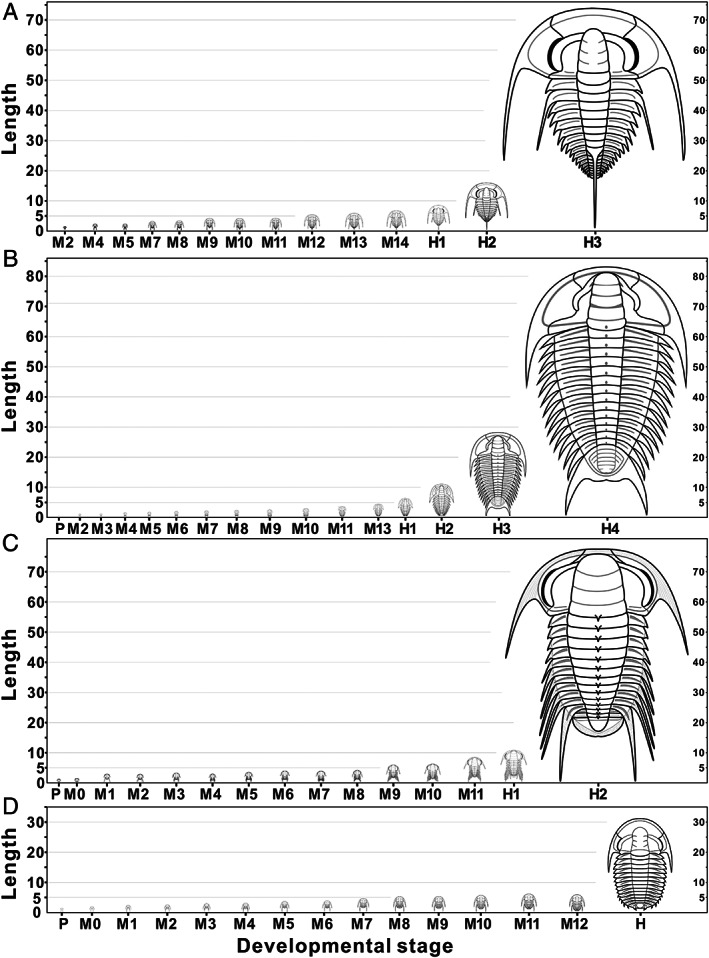
Reconstruction of the ontogenetic sequences of four redlichiid trilobites, showing the large differentiation in growth rate and in maximum exoskeletal length between the pre‐holaspid and holaspid phase. (A) *Eoredlichia intermediata*, modified from Dai & Zhang (2013*b*). (B) *Zhangshania typica*, modified from Hou *et al*. (2017). (C) *Bathynotus kueichouensis*, modified from (Zhang *et al*., 2022). (D) *Estaingia bilobata*, modified from Holmes *et al*. (2021*b*).

**Fig. 10:**
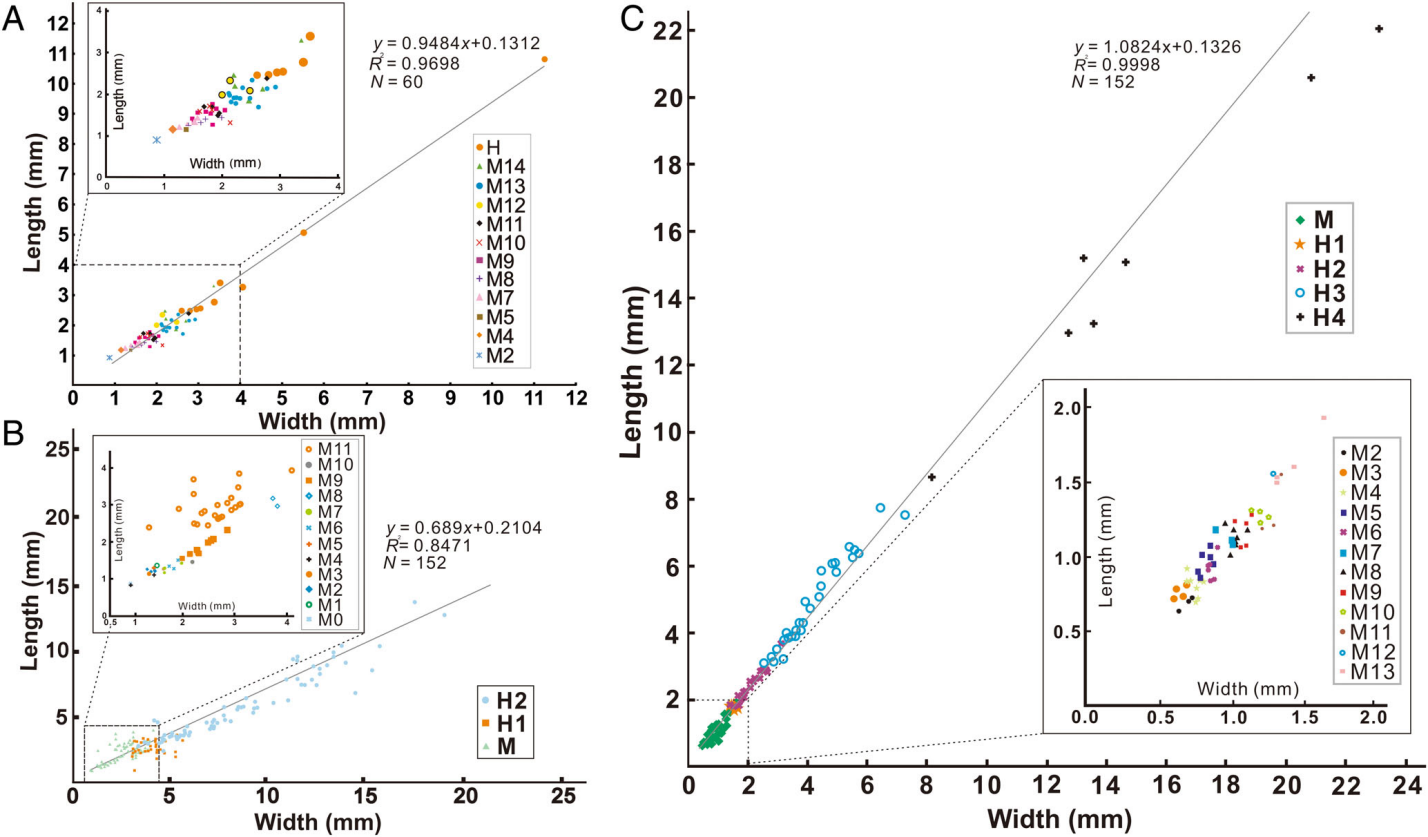
Isometric cranidial growth during the development of four redlichiid trilobites. Data for cranidial length are plotted against cranidial width for (A) *Eoredlichia intermediata* (data from Dai & Zhang, 2013*b*), (B) *Bathynotus kueichouensis* (from Zhang *et al*., 2022) and (C) *Zhangshania typica* (Hou *et al*., 2017). M, meraspid period; H, holaspid period.

The four redlichiids considered herein displayed not only a similar segmentation pattern during development, but also a large disparity in the size distribution between the pre‐holaspid and holaspid periods. The plots of developmental stage *versus* specimen length show that the size ranges of adults in these species are much broader than those of the protaspid and meraspid phases combined (Fig. 8). In *E*. *intermediata*, body length ranges from 0.96 to 5.96 mm for meraspides and from 5.82 to 57.89 mm for holaspides (Fig. 8A); in *Z. typica*, these length ranges are 0.55–4.55 mm for protaspides and meraspides, and 4.53–85.0 mm for holaspides (Fig. 8B); in *B. kueichouensis*, 0.87–5.98 mm in protaspides and meraspides, and 4.66–63.55 mm in holaspides (Fig. 8C); in *E. bilobata*, according to Holmes, Paterson & García‐Bellido (2022), body length ranges from 0.73 to 4.92 mm in protaspides and meraspides, and from 5.04 to 31.34 mm in holaspides (Fig. 8D). During the holaspid phase, all these taxa show an extremely wide size range, with a maximum length up to 19 times greater than that at the end of the meraspid period (Fig. 9). Thus, we speculate that these redlichiids probably spent a small portion of their life cycles as meraspides before reaching a full complement of thoracic segments, and a considerable portion as holaspides, during which a series of moulting events occurred with only a few segments added into the pygidium. Plots of cranidial length *versus* width in *E*. *intermediata* (Fig. 10A), *B. kueichouensis* (Fig. 10B), *Z. typica* (Fig. 10C) and *E. bilobata* (see fig. 8 in Holmes *et al*., 2021*b*) show considerable overlap between meraspid degrees, without any distinct size clusters that can be identified.

On the contrary, a totally different exoskeletal size distribution between pre‐holaspid and holaspid phases has been widely recognized in other groups, such as the Cambrian oryctocephalids *Duyunaspis duyunensis* (meraspides 0.56–7.05 mm; holaspides 3.58–6.96 mm; Dai *et al*., 2017), *Oryctocarella duyunensis* [meraspides 0.65–10.20 mm; holaspides (D11) 8.04–8.55 mm; Dai *et al*., 2021*b*], *Duodingia duodingensis* (protaspides 0.47–0.56 mm; meraspides 0.52–4.74 mm; holaspides 3.00–6.01 mm; Hou *et al*., 2015), and *Oryctocephalus indicus* (meraspides 1.2–12.0 mm; holaspides 12.0–25.0 mm; Esteve *et al*., 2017); the Cambrian olenids, *Peltura scarabaeoides* [meraspides (M2–M10) 1.2–4.0 mm; holaspides 5.75–13.0 mm; Bird & Clarkson, 2003]; and the Silurian aulacopleurids, *Aulacopleura konincki* [meraspides (M4/D4–M21/D21) 1.72–23.57 mm; holaspides (D22) 13.32–28.81 mm; Hughes *et al*., 2017]. In these cases, the size range of holaspid specimens is much more similar to that of the pre‐holaspid period, suggesting that the pre‐holaspides occupied a larger portion of the lifespan in which segment addition occurred, while the holaspid phase occupied a proportionately smaller part of the life cycle in which a few trunk segments were inserted into the pygidium. In these taxa, accordingly, a large proportion of their life cycles was involved in segment addition (i.e. the anamorphic phase), and a smaller proportion in the epimorphic phase before death. This contrasts with the redlichiids considered herein, in which a relatively short phase of segment production and liberation was followed by a long period during which the addition of segments to the thorax ceased and the rate of new segment expression was much lower than in the pre‐holaspid period.

### (2) Developmental strategy

From our description of the redlichiid segmentation pattern summarized in Section II, a 1:1 segment growth pattern may have allowed the animal to reach a full complement of thoracic segments in a relatively small portion of its lifespan. Therefore, these redlichiids may have taken less time to complete the addition of thoracic segments and used a relatively short meraspid period mainly for segment increase, followed by a longer holaspid phase mainly for increases in body size. This could perhaps have enabled them to adapt quickly to their environment or perhaps to be predators of larger size at the top of the food chain. From another perspective, in order to reach a large body size rapidly, they employed a faster growth rate involving rapid segment increase and addition to the thorax to achieve maximum efficiency in the formation of the thoracic or trunk segments during a short pre‐holaspid period, followed by enlargement in sclerite size during consecutive ecdyses throughout their remaining extensive life history. This developmental strategy allowed these redlichiids to possess a more streamlined configuration with homonomous trunk segments, and reach large body sizes that may be advantageous in terms of efficiency in predation and in intraspecific competition. This offers insights into the nature of developmental control and growth rates in these primitive arthropods, and may be significant in understanding the origins of arthropod body plans. The developmental pattern of a rapid and steady increase in trunk segment formation (especially in the thoracic region), which is unique to micropygous redlichiids, we here term ‘Lu's ontogenesis’, in honour of Prof. Yanhao Lu′s contributions to trilobite systematics and stratigraphy, as well as his first documentation of a redlichiid ontogenetic sequence from China.

The adults of some other trilobite groups are known to have multiple morphs with marked variation in the number of thoracic segments, which perhaps represents an alternative life strategy. For example, due to variations in the growth rate at the transition between the meraspid and holaspid phase, five adult morphs of *A*. *konincki* have been described with 18–22 thoracic segments (Hughes *et al*., 1999, 2017; Fusco *et al*., 2014), and adult *D. duyunensis* (Dai *et al*., 2017) and *O. duyunensis* (Dai *et al*., 2021*b*) are thought to have 8–10 and 9–11 thoracic segments, respectively. Such variations in adult segment number, which could be produced by irregular alternations between segment accretion and stasis (Fusco *et al*., 2004), are known not only in post‐Cambrian taxa, but also in many Cambrian early‐diverging clades.

Moreover, multiple instars per meraspid degree (defined by different pygidial segment numbers within the same degree) have also been observed in various early‐diverging clades, such as eodiscinids (Dai & Zhang, 2011, 2013*a*; Cederström *et al*., 2009; Zhang & Clarkson, 2012), oryctocephalids (Hou *et al*., 2015; Dai *et al*., 2016, 2017; Du *et al*., 2020), cheiruroidids (Dai, Zhang & Peng, 2014), and ptychopariids (Shen *et al*., 2014). This condition might also be present in some derived species such as *A. koninckii* in which variation in pygidial segment number (3 and 4) during ontogeny is also known in different meraspid degrees (see Hughes & Chapman, 1995). Multiple instars in each meraspid degree, which might be caused by intraspecific variation or individual differences in the rate of somitogenesis and tagmosis, suggest that they could build their body structures with some flexibility through different developmental trajectories (Dai *et al*., 2019). This pattern has not yet been observed in any redlichiid taxa. Holmes *et al*. (2021*b*) argued that the additional instars were highly unlikely to be found in each meraspid degree from M0 to M12 of *E. bilobata*, and that each meraspid degree represents a separate instar of a single developmental pathway. This developmental strategy with stability in somitogenesis and tagmosis during the meraspid phase may result in very low intraspecific variation in the number of trunk segments, perhaps explaining why the multiple adult morphs known in many polymerid trilobites have not been identified in redlichiids to date.

### (3) The distribution of specimen numbers at different growth stages

The number of specimens from different developmental stages can also provide evidence for developmental traits. The available material for *B. kueichouensis*, *E*. *intermediata*, *Z. typica* and *E. bilobata* has only a small number of protaspid and meraspid specimens, with the great majority being holaspides and covering a wide size range (Figs 8 and 9). This pattern is very similar to that of many eodiscoids for which ontogenetic sequences have been documented. For example, in the collections of *Tsunyidiscus* (Dai & Zhang, 2011; Dai *et al*., 2016), *Sinodiscus* (Dai & Zhang, 2013*a*) and *Pagetia* (Dai *et al*., 2019), there are usually hundreds to thousands of articulated and disarticulated holaspid specimens, but only a few dozen meraspides. This distribution is in direct contrast to that of other polymerid trilobites. For example, in collections of the oryctocephalid *O*. *duyunensis* (Dai *et al*., 2021*b*), there are hundreds to thousands of articulated meraspides but only a few articulated holaspides, and a similar bias is evident for *D*. *duyunensis* (Lei, 2016; Dai *et al*., 2017). A larger number of meraspid specimens encompassing a wider size range might suggest that these trilobites spent a longer period as meraspides and underwent more moulting events as meraspides than as holaspides.

In the latter case, the scarcity of holaspid specimens usually makes it difficult to find them during fieldwork. For example, for the cheiruroidid *Hunanocephalus ovalis* (Dai *et al*., 2014), more than 128 articulated specimens are known with thoracic segments numbering from zero (M0) to 10 (M10), with all assigned as meraspides because holaspides of this species were reported to have 12 thoracic segments (Zhang *et al*., 1980). Similarly, for *D. duodingensis* (see Hou *et al*., 2015), which has a similar morphology and close affinity to *H. ovalis*, its holaspides were reported to possess nine thoracic segments but showed little difference in morphology and size range from those of late meraspides (M8). Consequently, we suspect that these specimens assigned as holaspides might actually be meraspid degree 9.

Although these data concerning the size distribution of fossil material might be affected by taphonomic or collection bias, and accordingly cannot be considered as rigorous biological evidence, they do support our conclusions regarding the development pattern and growth strategy of these redlichiids: a short meraspid period occupying a small proportion of the life cycle during which the segment expression and transition from caudal plate to thoracic region occurred, with a subsequent lengthy holaspid period in which the rate of somitogenesis declined or ceased but body size increased rapidly.

## IV. CONCLUSIONS

(1) The four redlichiids considered herein used a relatively short meraspid period principally for trunk segment accumulation and allocation to the thorax in a balanced ratio. During a subsequent extended holaspid phase, segment release into the thorax ceased and new segment production decelerated, with this part of the life cycle involving increases in body size. This growth pattern with an equilibrium phase principally for segment ‘production and transition’ followed by an extended phase mainly for body increase may be general for the redlichiid group with micropygous body patterning.

(2) Variation in the rate of segment production and release, which is usually explained as evidence of intraspecific variation, has been found in various trilobite groups. However, this type of variation in thoracic and pygidial segment number during ontogeny has not been identified in redlichiids. This may be due to their development pattern, which may represent a more primitive segmentation condition that was modified by later clades. The various developmental modes in different groups are likely to represent strategies to regulate segment construction as an adaptation to their environment or functional requirements.

(3) The available material showing a complete developmental sequence for Cambrian redlichiid trilobites with a micropygous body plan is currently too limited to allow a full understanding of trilobite development. Since trilobite post‐embryonic development offers a unique window into the evolution of post‐cephalic body patterning within a major arthropod clade, it is important to continue to explore the variable nature of tagmosis within the trilobite trunk region and developmental evolution among early arthropods (Hughes, 2003*a*,*b*; Minelli, Fusco & Hughes, 2003). In the future work, an expanded data set including a greater diversity of high‐quality ontogenetic series will allow these hypotheses to be tested with respect to trilobite growth patterns. We believe that such studies will improve our knowledge on the essential attributes and traits of stem trilobitomorph taxa, and give trilobites a unique significance in the context of early arthropod evolutionary processes.
